# Using Lumbar X-Ray to Facilitate Modified Taylor’s Approach of Spinal Anesthesia in an Elderly Patient With Scoliosis

**DOI:** 10.7759/cureus.12556

**Published:** 2021-01-07

**Authors:** Gregory A Kirby, Wenjuan Guo, John D Mitchell, Haobo Ma

**Affiliations:** 1 Anesthesiology, Critical Care, and Pain Medicine, Beth Israel Deaconess Medical Center, Harvard Medical School, Boston, USA; 2 Anesthesiology, Peking Union Medical College Hospital, Beijing, CHN

**Keywords:** lumbar x-ray, scoliosis, taylor’s approach, x-ray guided, elderly patient, neuraxial anesthesia, spinal anesthesia, modified taylor's approach, geriatric patient

## Abstract

In geriatric patients scheduled for hip or knee surgery, neuraxial anesthesia is a safe and effective anesthesia method and may be a better option than general anesthesia. Unfortunately, establishing neuraxial anesthesia is not always easy in this group of patients. Anatomical abnormalities, such as spinal stenosis, scoliosis, and narrowed interspaces, contribute to the difficulties that anesthesiologists face while performing these procedures. The classic Taylor’s approach targets the widest interspace, L5-S1, as the needle insertion site and accordingly has an increased success rate in difficult neuraxial anesthesia. As this technique historically relies solely on palpation, it might be difficult in patients with less prominent or distorted anatomic landmarks. Ultrasonography or fluoroscopy guidance may help to better target the epidural or subarachnoid space, but both have limitations due to equipment availability or provider expertise. The modified Taylor’s approach we propose in this case report is based on preoperative lumbar x-ray interpretation when point-of-care image guidance cannot be performed. By measuring on the patient’s preoperative lumbar x-ray, we successfully performed a modified Taylor's approach of spinal anesthesia on an elderly patient with severe scoliosis. She underwent open reduction and internal fixation (ORIF) of the left femur with satisfactory pain control and no complications.

## Introduction

Neuraxial anesthesia is sometimes chosen over general anesthesia for lower extremity surgery. This type of anesthesia has proven to be as safe and effective as general anesthesia [1,2] and may even offer more favorable clinical outcomes compared with general anesthesia [3-6]. However, lumbar scoliosis, commonly seen in the geriatric population, poses a great challenge to neuraxial anesthesia placement. The L5-S1 level, being the widest interspace, is an advantageous entry site for needle insertion in difficult neuraxial anesthesia cases. It was first reported in 1940 and commonly known as the Taylor’s approach [7]. Additionally, utilizing image modalities such as ultrasonography or fluoroscopy can greatly increase the ease of completing epidural and spinal anesthesia. Both methods are effective but are limited by clinician's proficiency and equipment availability. Methodical interpretation of pre-existing preoperative lumbar x-rays can provide valuable anatomic information, but few studies or case reports have exploited this potential. Here, we present a case in which review of the preoperative lumbar x-ray facilitated spinal anesthesia through a modified Taylor’s approach in an elderly patient with severe lumbar scoliosis.

## Case presentation

A 90-year-old woman presented for open reduction and internal fixation (ORIF) of a left periprosthetic femoral shaft fracture. Five months prior, she underwent total knee arthroplasty complicated by periprosthetic femoral fracture corrected with ORIF. Following that ORIF repair, the patient suffered another non-traumatic periprosthetic femoral fracture. Her medical history was significant for atrial fibrillation, heart failure with preserved ejection fraction, hypertension, hyperlipidemia, lumbar spinal stenosis, and lumbar scoliosis. She was not on anti-coagulation therapy, and there was no known contraindication for neuraxial anesthesia. After weighing the risks and benefits of spinal anesthesia versus general endotracheal anesthesia, the patient opted for spinal anesthesia. 

**Figure 1:**
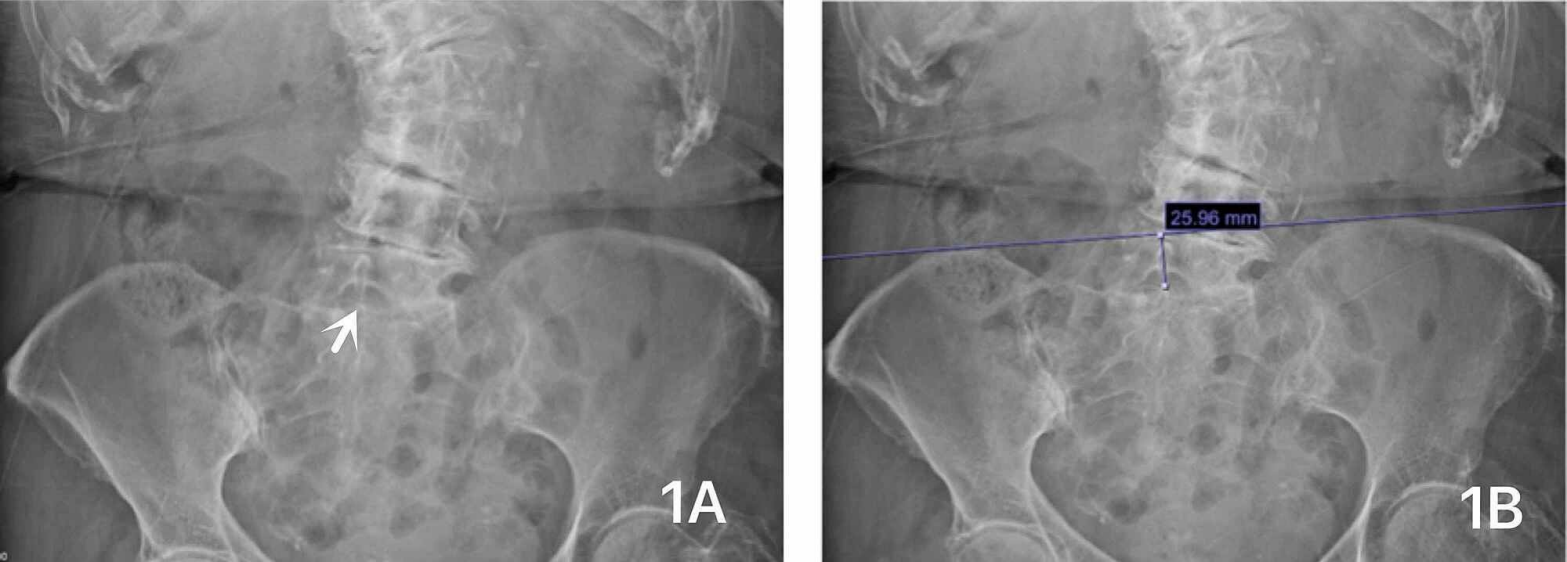
Patient’s lumbar x-ray. Figure 1A shows large radiographically patent L5-S1 interspace (arrow) with notable severe lumbar scoliosis, and Figure 1B shows the measurement of the distance between iliac crests and L5-S1 interspace.

In the operating room, she was positioned in right-lateral decubitus position after adequate pain control. Palpation confirmed the extent of her lumbar scoliosis. Preoperative lumbar x-ray showed L5-S1 as the widest patent interspace (Figure 1A). The distance between the iliac crests and the center of L5-S1 interspace was measured directly on preoperative lumbar x-ray as 2.6 centimeters (Figure 1B). We palpated her iliac crests and marked on the skin to indicate the L5-S1 interspace approximately 2.6 centimeters caudal to the level of the patient’s iliac crests. The skin was topicalized with 1% lidocaine, followed by placement of a 22-gauge Sprotte needle (Teleflex Inc., Wayne, PA) into the intrathecal space, which was confirmed by free flow of clear cerebral spinal fluid. Then, three milliliters of isobaric 0.5% bupivacaine were injected intrathecally. A T10 level sensory block was obtained prior to incision. A simple facemask delivered 4 liters per minute of oxygen while sedation was achieved with 40 mcg/kg/minute propofol infusion.

The surgical procedure lasted 121 minutes. The patient was hemodynamically stable on a 0.3 mcg/kg/minute phenylephrine infusion. She remained sedated throughout the surgery but was easily arousable to voice. The estimated blood loss was 250 milliliters. On arrival at the post-anesthesia care unit, she was alert and oriented. Her postoperative course was uneventful, and she was discharged on postoperative day two to a skilled nursing facility for continued physical rehabilitation. She had excellent recovery at her one-, two- and three-month postoperative follow-up and was back to her ambulatory baseline.

## Discussion

Neuraxial anesthesia (spinal, epidural, or combined spinal-epidural anesthesia) is widely used for surgeries involving the lower limbs, pelvis, or perineum. This type of anesthesia is equally effective when compared with general anesthesia [1,2] and offers favorable outcomes by decreasing respiratory complication and surgical stress response, reducing exposure to blood product transfusion, enhancing functional recovery, and shortening the length of hospital stay [3-6]. These outcomes are clinically beneficial for geriatric patients. 

The desired level of needle insertion in neuraxial anesthesia is typically identified by palpating the patient’s surface landmarks. However, scoliosis in the elderly can make the needle placement challenging [8-10]. Unfortunately, multiple insertion attempts at establishing neuraxial anesthesia may lead to nerve injury, hematoma, postdural puncture headache, infection, or inadequate analgesia [9]. After ensuring favorable patient positioning, the anesthesiologist may try the convex-side paramedian approach or convex-side angulation midline approach to ease the epidural or spinal needle placement, but the success rate for these techniques varies [8]. Several other approaches have been developed to improve the success rate and patient safety. One technique involves changing the needle insertion site to a wider interspace, such as the L5-S1 interspace used in the Taylor’s approach. Other approaches use point-of-care image guidance such as ultrasonography or live fluoroscopy.

The Taylor’s approach, or the lumbosacral approach, is neuraxial anesthesia performed at the L5-S1 interspace [7]. An L5-S1 spinal anesthesia is effective for a wide range of pelvic, perineal, and lower limb surgeries and has potential benefits including decreased incidence of hypotension [11]. Because L5-S1 interspace is the largest interspace in most patients, an approach at this level can be utilized when other approaches are unsuccessful or cannot be performed, like in patients with short statures [12], prior history of spine surgery [13], ankylosing spondylosis [14], or severe scoliosis [15]. The classic Taylor’s approach relies on palpation of the posterior superior iliac spine to guide needle insertion. This is sometimes difficult in patients with high body mass index, positional limitation, or abnormal anatomical surface landmarks [16].

Ultrasonography or fluoroscopy can offer valuable information on insertion site selection for neuraxial anesthesia. Direct ultrasonographic visualization of the interspace has been successfully used to aid neuraxial anesthesia for the obese, elderly patients, or patients with abnormal anatomical surface landmarks. Ultrasonography enhances precision and efficacy for needle insertion [17,18], increases first-attempt success rates, and reduces needle insertion attempts. Point-of-care ultrasonographic guidance has limitations pertaining to equipment availability and provider experience [18]. In cases when visualization is not adequate with ultrasonography, fluoroscopy is sometimes used. Live fluoroscopy is even more limited than ultrasonography as it relies on the availability of equipment and trained operators [19,20].

To overcome the limitations of point-of-care ultrasonography and fluoroscopy, we propose using pre-existing preoperative imaging, such as lumbar x-ray, instead of live ultrasonography or fluoroscopy. This is a modified Taylor’s approach, and it only requires a lumbar x-ray that is readily accessible in patients scheduled for hip surgery. The preoperative lumbar x-ray can relate the distances between the L5-S1 interspace and landmarks on the patient that might be more easily palpable such as the iliac crests. Certain artifacts such as bowel gas can interfere with interpretation, and referencing a lateral lumbar x-ray in addition to the anteroposterior lumbar x-ray can improve interpretation accuracy. The measurements obtained from preoperative imaging may not precisely transfer due to changes in the patient’s position at the time of neuraxial anesthesia placement compared to time of imaging study, possibly limiting the utility in some instances. Despite potential limitations, combining Taylor’s approach with the measurements obtained from preoperative lumbar x-ray images may increase the rate of successful neuraxial anesthesia placement.

There is currently limited published data on alternative neuraxial anesthesia placement techniques in patients with scoliosis. We presented our case of using available preoperative lumbar x-ray imaging to facilitate a modified Taylor’s approach of spinal anesthesia as an alternative to achieving safe and efficient neuraxial anesthesia placement in this patient group.

## Conclusions

When placing neuraxial anesthesia in patients with scoliosis, using preoperative lumbar x-ray images can help identify L5-S1 interspace and increase the success rate of the classic Taylor’s approach. Compared with the use of point-of-care ultrasonography or live fluoroscopy, review of preoperative lumbar x-rays for this modified Taylor’s approach can be easily accomplished and less limited by either equipment availability or trained operators.
